# Cardiology and oncology: a meeting of giants

**DOI:** 10.1590/1806-9282.2024S114

**Published:** 2024-06-07

**Authors:** João Pedro Passos Dutra, Ariane Vieira Scarlatelli Macedo, Tania Felix Lorenzato Fonseca Peixoto, Juliane Dantas Seabra Garcez, Bruno Cesar Bacchiega, Pedro De Marchi, Alexandre Manoel Varela, Bianca Jaccoud Amaral Martins, Carolina Maria Pinto Domingues de Carvalho e Silva, Renato Delascio Lopes

**Affiliations:** 1 Centro de Pesquisas Oncológicas (CEPON), SOS Cardio Hospital – Florianópolis (SC), Brazil.; 2 Faculty of Medical Sciences of Santa Casa de São Paulo, Americas Health Group – São Paulo (SP), Brazil.; 3 Hospital Felício Rocho – Belo Horizonte (MG), Brazil.; 4 Hospital São Lucas Rede D’Or São Luiz – Aracaju (SE), Brazil.; 5 Barretos Cancer Hospital – Barretos (SP), Brazil.; 6 Oncoclínicas – Rio de Janeiro (RJ), Brazil.; 7 Universitário Mackenzie, Curitiba Hospital, Erasto Gaertner Hospital – Curitiba (PR), Brazil.; 8 Centro de Pesquisa Oncológica, SOS Cardio Hospital, Florianópolis Specialized Oncology Center – Florianópolis (SC), Brazil.; 9 Brazilian Clinical Research Institute – São Paulo (SP), Brazil.; 10 Duke University, Duke Clinical Research Institute, School of Medicine – Durham (NC), United States.

## INTRODUCTION

The oncology field has experienced a revolution in recent decades. The ability for early diagnosis, associated with the emergence of various life-extending treatments, has reduced mortality rates for several neoplasms^1^. As a result, cancer often needs to be treated as a chronic disease that coexists with cardiovascular conditions. This advancement, coupled with the significant increase in cancer survivors, redefines the interdisciplinary relationship between oncology and other medical specialties. Given the need to enhance cardiovascular care for individuals who have or have had cancer, cardio-oncology has emerged as an exemplary area of this synergistic collaboration with oncology^2^.

Cardio-oncology is not limited solely to the study of the adverse effects of oncologic treatments. Instead, it encompasses a broader perspective on all possible interactions between cardiology and oncology^3^. In this context, we can highlight reverse cardio-oncology, which studies the intricate relationships between cardiovascular diseases and cancer^4^. In addition to aging, a range of modifiable risk factors, such as high blood pressure, diabetes, smoking, obesity, and a sedentary lifestyle, have a bidirectional relationship with the onset of cardiovascular and oncological diseases^5^. It is observed that oncology patients, following the oncological diagnosis across various primary sites, are more likely to die from cardiovascular diseases than the general population throughout follow-up^6^. Particularly in the older population and across multiple types of cancer, cardiovascular mortality can surpass cancer-related mortality over the follow-up period for these individuals^7^. In the postmenopausal women with hormone receptor-positive breast cancer subgroup, cardiovascular mortality is reported as the primary cause of death 8 years after diagnosis^8^. Furthermore, in surviving patients, cardiovascular events are associated with a higher likelihood of oncological disease recurrence^9^. On the other hand, individuals with cardiovascular disease are considered at higher risk of developing cancer, even when excluding conventional factors associated with atherosclerosis and cancer simultaneously^10^. Evidence from observational studies has shown an association between heart failure and an increased risk of cancer, highlighting the importance of prevention measures and early oncological diagnosis in this population^11^.

It is important to note that individuals with significant cardiovascular diseases are generally excluded from oncological clinical trials, and similarly, individuals with cancer are excluded from cardiology-related trials^12^. Thus, although there is significant overlap between these two specialties, there are many gaps regarding the optimal management of individuals with overlapping cancer and cardiovascular diseases, and we still lack robust evidence in this population. Therefore, it becomes essential to foster collaboration between these two fields, focusing on scientific research to elucidate the intersections between these areas, enhance cooperation, and improve communication among the involved professionals to provide better patient care.

## CARDIOLOGY: AN OLD GIANT

The history of cardiology is marked by significant developments throughout the 20th century. Examples include the development of the electrocardiogram, coronary care units, cardiac surgery, thrombolysis, cardiac catheterization, and coronary angioplasty, all of which drastically transformed the treatment of cardiovascular diseases. The Framingham study in the late 1950s was a milestone in cardiology research as it demonstrated the association between risk factors such as high blood pressure, dyslipidemia, and smoking and the development of atherosclerosis and major cardiovascular events. In modern times, it is known that these same factors are also related to the onset of cancer^13,14^.

With the advancement of knowledge in cardiology, whether in understanding common risk factors and overlap with various diseases, a significant interaction with other medical specialties has been observed. This interaction led to new subspecialties, such as cardiometabolism and cardio-oncology^15^.

## ONCOLOGY: AN EXPANDING GIANT

Oncology is a rapidly expanding field of medicine. The history of its development demonstrates a significant evolution in understanding the mechanisms related to the onset of cancer, coupled with the continuous development of new therapies^16-18^. Despite advancements in new cancer treatments and diagnostic methods, the number of individuals affected by oncological diseases worldwide remains enormous.

After the epidemiological transition, particularly in the second half of the 20th century, cardiovascular diseases and cancer emerged as the leading causes of mortality. Based on current trends, it is considered that cancer will surpass cardiovascular diseases as the primary cause of mortality in most countries in the following years^19^.

Over time, the mainstays of cancer treatment have been surgery, chemotherapy, and radiation therapy. However, in recent years, targeted therapies have played a prominent role in research in the pursuit of greater precision regarding the action of drugs against specific proteins and genes related to cancer^20^. These interventions targeted at specific sites have altered the course of numerous oncological diseases, with imatinib emerging as a pioneering example^21^. Furthermore, a better understanding of the immune system and its interactions with cancer has also positioned immunotherapy as a critical player in many oncological treatments^22^. Therefore, molecular therapy, cellular therapy, immunotherapy, metabolomics, proteomics, and various genetic markers have been the cornerstones of precision medicine in the oncology field. These personalized approaches support guided medical decisions, allowing treatments to be more effective and with fewer adverse events^23^. One of the current challenges is the implementation of and increased access to precision medicine^24^.

## ONCOLOGY AND THE PANDORA’S BOX

Oncological treatments can cause toxicities in various forms. The discovery and application of innovative therapies are associated with growing concerns about new side effects. Advancing the field with novel and particular treatments is always challenging because one needs to learn how to manage unknown and unexpected adverse clinical outcomes in real time.

The ideal scenario where targeted therapies can affect only cancer cells has not yet been achieved. To illustrate, we can mention the adverse effects of tyrosine kinase inhibitors (TKIs), which can occur in two models: 1. "on-target" toxicity, where the inhibited molecular target plays a crucial role in tumor proliferation and normal cell survival pathways and 2. "off-target" toxicity, that results from the action of TKIs on other targets that are unrelated to their antitumor activity, more familiar with multitarget inhibitors, such as sunitinib^25^.

Regarding cardiovascular adverse effects, there is a spectrum regarding the severity of toxicities. For example, the new immunotherapy drugs (immune checkpoint inhibitors, or ICIs), which have rapidly expanded their indications in various oncological treatment scenarios (curative, palliative, and adjuvant), present situations like immune-mediated myocarditis. Although the incidence of this condition is very low, it carries a high mortality rate^26,27^. Therefore, with the emergence of numerous oncological therapies, it becomes crucial to learn, identify, and manage the specific complications of each drug, ensuring that these events do not hinder the continuation of such promising oncological treatments^28^.

## CARDIO-ONCOLOGY AND THE MYTH OF SISYPHUS

Cardio-oncology emerged in the 1970s when cardiac damage related to chemotherapy drugs, specifically anthracyclines, was observed^29^. The findings were based on myocardial biopsy analysis, considering today's imaging methods are not yet accessible^30^. However, cardio-oncology regained attention following the introduction of trastuzumab treatment for breast cancer. In the first study that combined anthracycline with this anti-human epidermal growth factor receptor 2 (HER2) monoclonal antibody, high rates of cardiotoxicity were observed, leading to the understanding that these medications cannot be used concurrently due to their synergistic mechanisms of cardiotoxicity^31^. Since then, numerous treatments and possible cardiovascular complications have emerged in recent years. With this demand, we have seen the expansion of cardio-oncology and the requirement for professionals involved in this field to understand the cardiovascular management of oncological patients^3^.

An illustration of the interaction between cardiology and oncology is the association between cancer and its treatments and coronary artery disease, a significant cause of mortality in cancer survivors^32^. In this context, it is noteworthy that some individuals may have coronary artery disease even without the four standard modifiable risk factors (high blood pressure, diabetes, dyslipidemia, and smoking). Global data mention that it can account for up to 11.6% of cases of acute coronary syndrome where no conventional risk factors are found^33^. Oncological treatments associated with the possibility of coronary artery disease as an adverse event should be included in this population's list of items to be evaluated^34^.

There is also a hypothesis that individuals with advanced cancer may exhibit a syndrome associated with heart failure related to various cancer-related factors, which would result in something called cardiac wasting, a degenerative form of cardiomyopathy linked to structural and electrical changes in the heart, leading, for example, to a higher risk of arrhythmias in these individuals^35^. This condition, independent of the adverse effects of oncological therapies, must be considered to identify patients with "cancer cardiomyopathy" so they can be promptly treated^36^. Therefore, the bidirectional relationship between cancer and heart failure motivates the study of the role of specific biomarkers in identifying individuals at higher risk of having existing cardiac alterations and a greater likelihood of cardiotoxicity with oncological treatments^37^.

To illustrate the complexity related to cardio-oncology, especially in cancer types where survival has significantly increased in recent years, such as breast and prostate cancer, we can draw parallels with the myth of Sisyphus. This king tried to cheat death and was condemned by Zeus to roll a stone uphill, only to watch it fall back down for eternity. Similar to this mythological story, physicians and healthcare providers who treat patients with oncology and cardiology complications often face a heavy burden and continuous challenges related to cancer and cardiovascular diseases^38^.

## CARDIOTOXICITY AND ITS CHALLENGES

Cardiotoxicity is defined as any cardiovascular impairment during or after oncological treatment, whether symptomatic or detected in complementary tests, after excluding other causes^39^. Therefore, we should understand that cardio-oncology deals with all cardiovascular diseases in the context of individuals with cancer. A point that poses difficulty in understanding cardiotoxicity is the need for more agreement among various medical societies regarding the definition of each specific cardiovascular condition. For example, there is significant variability in the left ventricular ejection fraction criteria that characterize an individual as having cancer therapeutics-related cardiac dysfunction (CTRCD) (Table 1)^3,40^. One of the initiatives that tried to unify different definitions systematically was the publication of the European Cardio-Oncology Guidelines in 2022, a comprehensive document serving as a guide for study and practice in the field^3^.

**Table 1:** Differences in published definitions of cardiotoxicity.

	Cutoff for left ventricular ejection fraction (LVEF)	Change in EF (ejection fraction) (absolute reduction)	Global longitudinal strain (GLS)
ESC 2022	Severe—new LVEF to <40% Moderate—new LVEF reduction by ≥10% to an LVEF of 40–49% Mild—LVEF o an LVEF of 40y ≥r ejection fracGLS by 15% from baseline	–	Moderate—new LVEF reduction by 10% to an LVEF of 40–49% and either new relative decline in GLS by 15% from baseline Mild—LVEF ≥50% and new relative decline in GLS by >15% from baseline
EACVI/ASE	<53%	>10% decline from baseline	Relative reduction in GLS >15% from baseline
ESMO	<55%	Decline ≥5% to less than 55% with symptoms or decline ne ≥5% to less thawithout symptoms	–
ASCO	<55%	–	Relative reduction in GLS >15% from baseline
CTCAE	<50%	Grade 2 (resting EF 40–50%; 10–19% drop from baseline); Grade 3 (resting LVEF 20–39%; >20% drop from baseline) Grade 4 (resting LVFE <20%)	–
FDA	–	>20% decrease if LVEF remained normal, or >10% decrease if LVEF is less than normal	–

ASCO: American Society of Clinical Oncology; ASE: American Society of Echocardiography; CTCAE: Common Terminology Criteria for Adverse Events; EACVI: European Association of Cardiovascular Imaging; ESC: Cardio-Oncology Council of the European Society of Cardiology; ESMO: European Society for Medical Oncology; FDA: US Food and Drug Administration; HFA: Heart Failure Association.

As mentioned earlier, a challenging aspect is the rapid emergence of many new oncology drugs in recent years. Pivotal oncological studies responsible for approving new therapies are typically done with a small number of patients, which limits the ability to determine possible adverse effects. Often, adverse effects are properly assessed after large-scale, real-world use of these therapies. Moreover, with the same speed at which treatments emerge, they can also become obsolete from an oncological standpoint. Therefore, if cardiologists take too long to determine the best way to deal with the cardiotoxicity of these drugs, this knowledge may become out of date. Thus, although basic and translational research has defined many pathophysiological mechanisms related to various forms of cardiotoxicity, best practices regarding monitoring and management are still to be studied in large-scale clinical trials^41^.

It is important to note that cardio-oncology generally bases its approaches on knowledge derived from general cardiology. This is the case of the management of cardiovascular conditions overlapping with oncological diseases, which are handled in the same way one would take patients without cancer. However, it is crucial to emphasize that there are many circumstances where these generalist approaches are insufficient^42^. To contextualize these situations, we should mention the concept of permissive cardiotoxicity—allowing the continuation of oncological treatment in a scenario of tolerable cardiovascular changes, establishing optimized clinical management, and frequent cardiac follow-up in conjunction with oncology. Thus, continuing oncological therapy is associated with increased survival and improved quality of life^43^.

Another example of an unexpected cardiac complication following cancer treatment is the appearance of atrial fibrillation related to using Bruton's TKIs, which are primarily manageable and do not necessarily require suspending oncological therapy^44^. Alternatively, in cases of hypertension related to vascular endothelial growth factor (VEGF) inhibitors, elevated blood pressure can occur rapidly after initiating these medications and reflect effective inhibition of VEGF signaling, which has been considered a biomarker related to tumor responsiveness. Therefore, in these situations, it is necessary to be vigilant for this joint adverse event and control hypertension to allow patients to continue treatment with VEGF inhibitors^45,46^.

It is crucial to go into the details of each therapy and the various conditions related to cardiotoxicity. The primary goal is to recognize that not all cardiovascular changes require treatment interruption. Permissive cardiotoxicity opposes the concept of prohibitive cardiotoxicity, where, due to a lack of knowledge related to cardio-oncology, there might be hasty recommendations to discontinue oncological therapies that could be essential for the survival of some individuals^43^. In HER2-positive breast cancer, for example, discontinuing treatment due to cardiotoxicity is associated with worse oncological outcomes^47,48^.

Additionally, we should emphasize the importance of imaging methods in the interaction between cardiology and oncology. Advanced imaging technology enables the early detection of cardiac changes in oncology patients, allowing for timely and personalized interventions^49^. The ability to critically interpret and understand the benefits and limitations of each exam, such as the details related to intra- and inter-observer variability in the analysis of left ventricular ejection fraction on echocardiograms, is essential^50^. Ultimately, the main goal is to avoid the erroneous interruption of oncological treatments. In cardio-oncology, attention should be paid to preventing overscreening and overdiagnosis, which are related to the unnecessary use of complementary tests. Cardio-oncology guidelines present extensive recommendations guiding the frequency of biomarkers and imaging testing that seem excessive and challenging to implement in clinical practice^51^.

## CARDIO-ONCOLOGY SERVICES AND THE LESSONS OF HERMES

**Figure 1:**
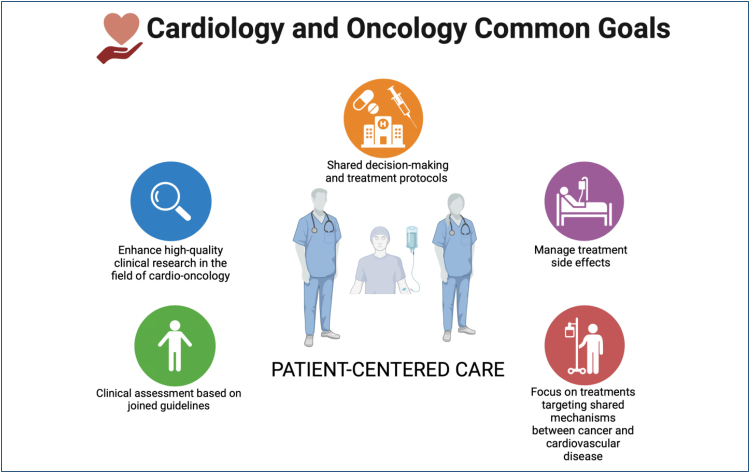
Cardiology and oncology—common goals.

The primary goal in treating cardio-oncology patients is to provide comprehensive, multidisciplinary, and integrated care so they can receive the best available oncological treatment with the highest possible safety (Figure 1). Therefore, the aim is to identify and treat pre-existing cardiovascular conditions and assist in risk assessment and monitoring to mitigate the potential adverse effects of oncological therapies^52^. There are several documents worldwide about the establishment and structuring of cardio-oncology services. In most of them, particular emphasis is given to strengthening communication^53-55^. To illustrate, we draw a parallel here with the lessons we can learn from the mythological god Hermes, an intelligent and clever entity responsible for communication between gods and mortals. Therefore, fostering better communication is crucial in various aspects and among all stakeholders involved with cardio-oncology.

The care of oncology patients is a complex endeavor that emerges from collaboration among various medical specialties and all healthcare providers. The intersection between cardiology and oncology also highlights the need for a patient-centered, multidisciplinary approach and the individualization of decisions. Another point is to encourage patients to be active participants in their care by adopting habits associated with preventing cardiovascular diseases and assisting in oncological treatment^56^. Furthermore, allowing patients the space to express their viewpoints in medical conferences, for example, it is important to note that oncology patients, especially those with overlapping cardiovascular diseases, can experience significant psychological impact. Therefore, we should have a broad perspective regarding the approach to this population, placing patient support and understanding their needs at the center of care. Thus, we emphasize that empathy, effective communication, and emotional support play a vital role in this process^57^. It is worth highlighting that these skills, known as soft skills, can and should be trained to improve the connections between physicians and patients^58^. Some points that can be mentioned in this regard are (a) prepare with intention: review the patient's history; (b) listen intently and thoroughly: listen without interruption; (c) agree on what matters most: determine the patient's concerns and priorities; (d) connect with the patient's story: empathize; and (e) explore emotional cues: be attentive, elicit, reflect, and validate the patient's signals^59^.

The current need for more research in cardio-oncology and the generation of better evidence related to the management and monitoring of oncology patients is evident. Many decisions in cardio-oncology are based on limited evidence. It is worth noting that in the 2022 European cardio-oncology guideline, only 2.6% of the 272 recommendations were classified as level of evidence A, with more than 75% earning the lowest level of evidence C. Although this is frustrating, it also makes cardio-oncology an exciting and dynamic field with significant opportunities for the development of clinical studies. One way to enhance collaboration between the fields would be the partnership between cardiologists and oncologists in participating in and designing clinical trials to establish and analyze cardiovascular outcomes in an adjudicated manner, for example, as occurred in the Pronounce study^60^. Furthermore, for cardio-oncology to strengthen, more studies demonstrating the beneficial impact of specialized cardio-oncology care are needed^61^.

Academic training for healthcare professionals is another crucial aspect to consider. In Brazil, cardio-oncology still needs to have the status of a regulated subspecialty, but there are postgraduate courses recognized by the Ministry of Education (MEC). We should emphasize the importance of the International Cardio-oncology Society (IC-OS), an organization that, in addition to various educational activities, organizes the international certification exam for professionals dedicated to cardio-oncology.

The expansion and relevance of cardio-oncology in recent years are undeniable, both due to epidemiological issues and the complexity of cardiovascular care for oncology patients. Therefore, we should consider expanding the discussion with society and healthcare providers about the availability of more structured cardio-oncology services. In this debate, the focus should be on appreciating and integrating professionals with expertise in the field into the oncology patient's journey. Encouraging oncology clinics to consider excellent cardiovascular safety inpatient treatment as a mandatory point of service excellence is crucial. Additionally, for the development of cardio-oncology clinics, it is critical to observe the particularities of each center. This approach identifies structural possibilities and the main areas needing improvement in various oncology-related aspects.

## CONCLUSION

Cardio-oncology is still a new field in medical knowledge, with a growing number of publications and increasing recognition due to the significant interaction between cancer and cardiovascular diseases in a bidirectional relationship. Through collaboration and a profound understanding of the complexities of these conditions, we can offer patients a better quality of life and improve outcomes related to cancer and cardiovascular diseases. Everyone involved in cardio-oncology is responsible for seeking a better understanding of the balance between cardiovascular risk and the optimal management of cancer, aiming to minimize unnecessary interruptions in oncological treatments and mitigate effects related to cardiotoxicity. Advances in research, a dedicated focus on comprehensive educational programs, the promotion of better communication among healthcare professionals, and humanizing care are essential to pave the way for more precise and evidence-based approaches to treating oncology patients.
